# Graph Pangenomes Track Genetic Variants for Crop Improvement

**DOI:** 10.3390/ijms232113420

**Published:** 2022-11-03

**Authors:** Amir Hameed, Pawel Poznanski, Anna Nadolska-Orczyk, Waclaw Orczyk

**Affiliations:** Plant Breeding and Acclimatization Institute-National Research Institute, Radzikow, 05-870 Blonie, Poland

**Keywords:** breeding, cereals, climate change, heritability, genome, presence-absence variants, structural variants, trait

## Abstract

Global climate change and the urgency to transform crops require an exhaustive genetic evaluation. The large polyploid genomes of food crops, such as cereals, make it difficult to identify candidate genes with confirmed hereditary. Although genome-wide association studies (GWAS) have been proficient in identifying genetic variants that are associated with complex traits, the resolution of acquired heritability faces several significant bottlenecks such as incomplete detection of structural variants (SV), genetic heterogeneity, and/or locus heterogeneity. Consequently, a biased estimate is generated with respect to agronomically complex traits. The graph pangenomes have resolved this missing heritability and provide significant details in terms of specific loci segregating among individuals and evolving to variations. The graph pangenome approach facilitates crop improvements through genome-linked fast breeding.

## 1. Introduction

As the temperature rises beyond 1.5 °C, the emerging climate change regime will have a strong impact on terrestrial agriculture [1]. Staple food, in which a large proportion comes from cereals, needs urgent transformations to ensure food security and crop productivity [2]. Traditional breeding has no time to achieve the fast pace required to feed the upcoming human population. Fast breeding cointegrated with genomics has facilitated crop improvement in the last two decades; however, it remains a challenge for crops with complex polyploid genomes and linked agronomic traits [3]. Since the first reference draft of the plant genome around two decades ago [4], there are several genome assemblies available in the database for many crop plants that cover variable depths, sizes, and versions [5]. In a perfect situation, a reference genome provides information about the nucleotide sequences of genes on all chromosomes and serves as a catalog (an annotation) to describe the arrangement of genes in a specific order on the genome relative to chromosomes, noncoding sequences, and centromeres. However, most of the single reference genomes developed so far with short-read sequencing rely on gene space, i.e., the representation of coding regions (core genes) among noncoding sequences (dispensable genes) and lack comprehensive information about noncoding genomic regions (introns), structural variants (SVs), insertions/deletions (InDels), copy number variations (CNVs), presence–absence variations (PAV), transposable elements (TE) and other single nucleotide polymorphisms (SNPs) that arise during evolution. Not surprisingly, most of the regulatory genes involved during different stresses (biotic/abiotic) are within these dispensable regions of the genomes [6], and there is missing information that needs to be filled in. Advancements in nucleotide sequencing technology, such as PacBio, HiFi-Seq, Oxford Nanopore, MGI nanoball, and Hi-C scaffolding, along with improved algorithms and computing power, have enabled the construction of pangenomes of different crop species. Pangenome, in a simple way, is a collection of high-quality reference assemblies of a particular species combined to make a standard reference assembly (covered in several reviews [7,8,9,10]). The next phase of this evolutionary road is the construction of graph pangenomes (an improved graphical visualization) that address new insights into genomic depth and breadth [11]. The dissected genetic core (conserved genes) identified through the graph pangenome could serve as a platform for fast forward breeding linked with the selection of beneficial alleles for genome editing (GE) or marker-assisted selection (MAS). Therefore, emerging pangenomics has the potential to improve the breeding of food crops with complex agronomic traits.

Genome-wide association studies (GWAS) predict the inheritance of complex biological traits; however, in many cases, this approach is limited by overlooking a significant portion of genomic variation. For example, the inability to detect SVs in a genome assembly mapped against a single reference genome leads to linkage disequilibrium between genomic loci and causal SVs. Furthermore, allele heterogeneity and locus heterogeneity are less captured due to the lower statistical power of GWAS resilient on mapping based on a single reference genome. Thus, genetic variants in the form of TE, InDels, CNVs, PAV, and chromosomal rearrangements remained underestimated while using a single reference genome. To improve and resolve these constraints in GWAS, a precise and comprehensive catalog is required and could be achieved by constructing a graph pangenome [12]. The applications of the graph pangenome to find the SVs that might be used for targeted GE and fast-breeding of crops are the main topic of this brief review. We tried to provide a critical assessment of graph pangenomes published so far for crops such as tomatoes, wheat, cucumbers, potatoes, and soybeans. In addition, we discuss the off-limit potentials of graph pangenomes to dissect genomic variants for trait improvements, such as disease resistance and quantitative yield attributes, in crop plants. Next, we propose the prospects of this technology for some beneficial outputs in plant science.

## 2. The Transition from Linear High-Quality Reference Genome to Graph Pangenome

In theory, a reference genome is built to provide a standard for comparison to genomes of other organisms within that species or related species. However, so far no boundaries have been defined so far about an “ideal reference genome” and the notion varies between geneticists and molecular researchers depending on their goals to utilize the genomic data [13]. Genome assemblies previously built with short-read sequencing are inefficient in covering the complexity and size of many plant genomes. Still, they have significance in generating hypotheses for gene evolution, ontology, and selecting markers for crop selection. With the advancement in next-generation sequencing with gradual lowering costs and increasing availability of long-read data, today an ideal reference genome may be represented by a pangenome at a further step [14]. The availability of a high-quality genome assembly covering chromosomal or haplotyping scaffolds for complex genomes provides new foundations in genomics for GWAS and other analyses. Pangenomes for many crops are becoming accessible providing comprehensive chromosome-level information that could be used to study SVs, gene evolution, duplications, and gene loss within a species or across the species. The shift from a high-quality reference assembly to a pangenome not only reduced the bias to detect SVs and other evolution events but also provided an ideal standard reference to fill the missing gaps in genomics. For example, two decades ago in 2012, the first draft genome of rice (*japonica* and *indica*) was published, and very recently in 2022, the rice pangenome was published [15]. The rice pangenome explored the SVs in rice and improved our knowledge of rice genomics. The authors described a deletion in a candidate gene that directly affected gene expression patterns in Asian rice and exhibited varied phenotypes with grain weight [15]. The inclusion of wild species in the pangenome has good practical value to explore the origin of domestication and trait mapping for crop improvement that otherwise would not have been possible with a single reference genome.

**Figure 1:**
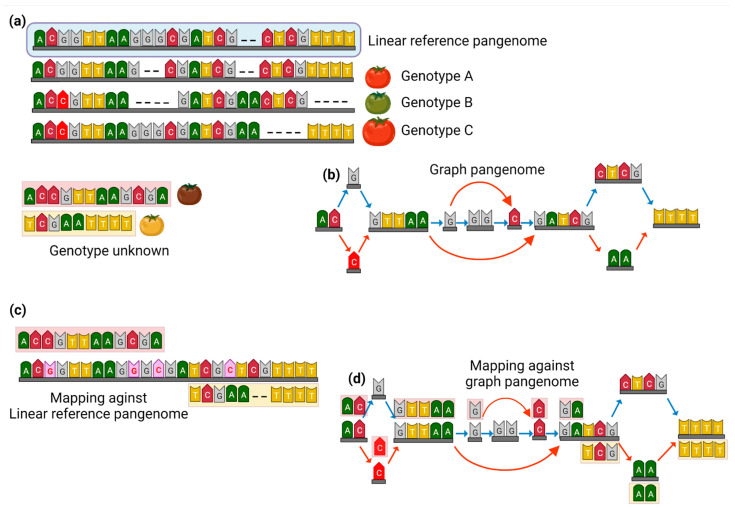
Schematic model of the graph pangenome that enhances the mapping efficiency of unknown genotypes against a reference genome. (**a**) A linear reference genome is constructed by aligning the mapped sequences of multiple accessions; (**b**) a graph pangenome is constructed from the aligned sequences, the red arrows (edges) show the variation in genotypes and provide additional paths for mapping against conserved regions (nodes); (**c**) mapping of unknown genotypes against linear reference pangenome may lead to mismatched and sequence gaps as information about variants is not ideally captured during this mapping approach; (**d**) graph pangenomes provide maximum coverage for mapping and avoid mismatches, and makes it possible to observe genetic variations with full-spectrum. For example, the unknown genotypes are mapped against graph-pangenome even having variants and gaps, and properly align to corresponding sequences through edges (red arrows).

The difficulty is comprehensible in light of the wealth of information that pangenomes generate [16]. Pangenomic models range from straightforward collections of unaligned sequences to visual representations. Graph pangenomes are becoming popular because they show accurate functional annotation of entire genomes portrayed in compressed data visualization while retaining the genomic diversity and inter/intra species variations (Schematically illustrated in Figure 1). Genomic sequences (nodes), their connections (edges), and details (paths) on how the nodes are arranged in each constituent genome make up a graphical representation of the pangenome [11]. There are several in silico tools available to construct the graph pangenome with the inclusion of high-quality reference genome assemblies for variation detection [17]. For example, the *Variation Graph Toolkit* (VG) [18] can merge a single reference genome assembly with multiple genome assemblies to develop a graph orientation of the pangenome while showing variations among the template genomes. Likewise, *Minigraph* [19] is another computational tool for creating a graphical view of mapped genome assemblies, which can display SVs and other important features of pangenomes. The lack of appropriate bioinformatic tools is the biggest barrier to mainstream implementation of pangenome graphs, because those made for linear genomes are difficult to transfer into graph orientation. Famous tools for linear pangenomics include *VG map*/*Minigraph* for mapping and alignment, *Graphtyper* for variant genotyping, *Paragraph* for SVs, *PLAST* for homology searches, *VG rna* for transcript mapping/quantification, and *Bandage*/*MoMI-G* for visualization (adapted from [9]). Integration of the graph pangenome with other molecular computing such as transcriptomics, metabolomics, and epigenetics makes an ideal dataset for further analysis and functional characterization of genomes.

## 3. Graph Pangenomes to Track Genetic Variability within Crop Plants

Exploring genomic diversity is important for identifying the footprints of gene loss/gain during evolution and for finding beneficial targets for improvements. Detection of SVs is one of the promising factors responsible for phenotypic differences within a species and responsible for variations in many important agronomic traits. Recently, Alonge et al. [20] discovered 238,490 SVs in tomatoes (100 accessions) leading to significant expression changes affecting fruit flavor, size, and yield. Interestingly, 10% of these SVs were detected in regulatory regions (dispensable genome), making a 1.4-fold change in gene expression as compared to 50% of SVs in exons contributing 2.5-fold change. This highlights the importance of SVs in determining the phenotypic variation in crop plants. The adaptation of crops to various agroecological zones facing new stresses could be traced through pangenomics datasets and could be exploited through pangenomics-assisted breeding.

Potato (*Solanum tuberosum* L.) is a highly diverse crop providing major food to the world. Current potato production is dominated by autotetraploid cultivars originally derived from wild diploid *Solanum* species. Recently, Tang et al. [21], to obtain an in-depth understanding of potato evolution and diploid breeding based on true seeds, constructed the potato pangenome by assembling long-read data of 44 diploid accessions of cultivated and wild tuber bearing, as well as 2 accessions of non-tuber bearing *Solanum* species. The pangenome approach enabled the discovery of novel tuberization factors, the discovery of another species, *Solanum candolleanum* as the immediate wild progenitor of cultivated potatos, and the identification of more than 57,000 nucleotide-binding domain and leucine-rich repeat (NLR) genes of the potato immune system. The former discovery indicated that tuber-based propagation enlarged a pool of disease resistance genes and significantly shaped the evolution of the potato genome [21].

**Figure 2:**
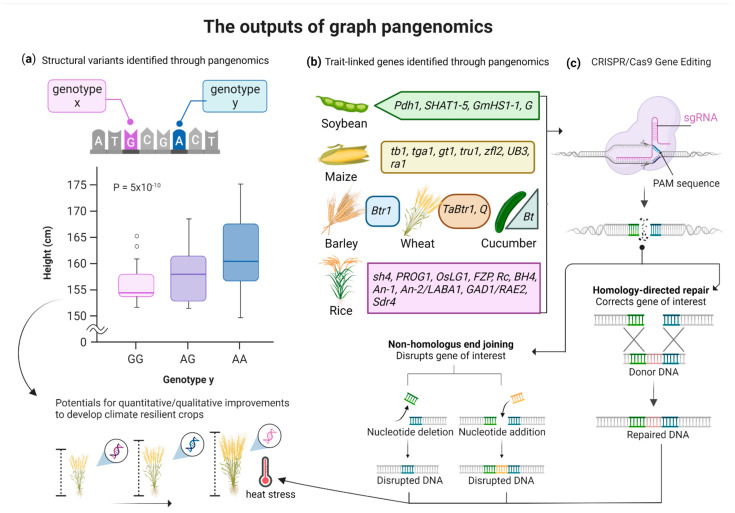
Schematic illustration of graph pangenomics outputs for crop improvements. (**a**) The analyzed datasets of graph pangenomes help identify structural variants among different genotypes that could be integrated after MAS into crops for trait improvements; (**b**) pangenomics identified trait-linked genes in different crops (adapted from [3]); (**c**) the identified gene targets could be used for fast-breeding using the CRISPR/Cas toolbox. This may bring desirable site-specific edits either through homology-directed repair or by introducing double-stranded breaks and subsequent cell DNA repair mechanisms, or by natural but the most time-consuming introgression breeding. Altogether, the identified traits or gene-edited plants may be developed to cope with ongoing climate change stress, such as temperature rises.

Recently, Zhou et al. [22] created a tomato graph pangenome to cover the missing heritability across multiple germplasms of red fruit tomatoes. The graph pangenome harnessed the potential of multiple reference genomes and integrated all the reference and the nonreference alleles into a single graph genome. In addition, it demonstrated better results compared to a single linear reference genome in calling distinct genetic variants such as SNPs, InDels, and SVs in various simulation studies. By employing novel genome sequencing technologies (HiFi and Hi-C), they assembled 32 reference-level long-read genome assemblies of tomato and combined these with other previously short-read assemblies (>806 accessions) to create a tomato graph pangenome (designated as TGG1.1). The main objective was to determine the genomic variations and to track the complex heritable traits within the species diversity. To do so, they mapped the genomic data of other 332 tomato accessions (Illumina sequences) against this TGG1.1 to find the genetic variants (SNPs, InDels, and SVs) linked with complex agronomic traits. As a control, they used a high-quality linear genome assembly (SL5.0) to compare the results of the missing heritability. Overall, a 24% higher genetic heritability (0.41 vs. 0.33) was observed with the inclusion of the graph pangenome that covered 20,323 molecular and metabolite traits. This was probably due to a higher mapping rate with a reference graph pangenome, where surplus genomic sequences are available from different accessions to cover the missing gaps. This enabled the tracking of missing heritability and identified more accurate linkage that otherwise was not detected in single reference-based mapping. Focusing on a single gene in tomato (Solyc03G002957) involved in phosphoinositide, they found an increased total heritability of 0.75 vs 0.54 in cis- and trans SVs. Another very recent study published the graph pangenome of cucumber and described SVs associated with agronomic traits [23]. By combining 12 high-quality genomic assemblies of different cucumber accessions, a graph pangenome was developed to map the large chromosomal rearrangements associated with warty fruits, flowering times, and root growth. The identified SVs comprising 3213–21,261 large InDels (mostly large insertions) across seven chromosomes of cucumber provide useful information on the hereditary and evolution of genomic variants. In addition to SVs, large inversions were detected in maize by pangenomics analyzing the high-quality genome assemblies of 66 inbred lines [24]. The datasets revealed large InDels and chromosomal inversions at multiple locations across the maize genomes, with a significant inversion of 75.5 Mb on chromosome 2. The re-inversion of this genomic segment with the CRISPR-Cas toolkit proofreads the practical application of pangenomics for precise editing of crop genomes. The discovery of SVs is valuable for GE and trait-assisted breeding programs. The output of graph pangenomes based on multiple reference genomes with hereditary information may help with the MAS of cereals for climate resilience (schematically illustrated in Figure 2).

## 4. Graph Pangenomics for Crops Improvement

The road to crop breeding for variety development started with MAS in the early 1990s [25], where genomic markers called QTLs (quantitative trait loci) were used for trait improvement. Next, the creation of reference genomes aided the process of finding candidate genes for genetic engineering and breeding programs and attempted to resolve SVs, InDels, CNVs and other SNPs with low resolution in GWAS [26]. The revolution in green biotechnology was fueled by the discovery of trait-associated alleles and genes. For example, the first GM tomato ‘FlavrSavr’ released in the 1990s [27] was a successful candidate that paved the way for the commercialization of many crops with improved/edited traits for biotic/abiotic stresses and to increase yield. In dealing with polyploid crops, the utility of a single reference genome is insufficient to cover the genetic complexity and find potential genes with confirmed heredity. Thus, pangenomes are in the field to explore genetic variation with full spectrum and enhanced efficiency and facilitate breeding programs. The utility of pangenomes to dissect regulatory genes that reside in the dispensable genome is another promising approach to developing stress-resistant crops (Table 1). Pangenome datasets resolved SVs detection and functional studies revealed that SVs are mainly responsible for differential gene expression regulated by *cis*-regulatory elements. Little variations in the *cis*-elements of promoter regions of genes lead to the structural reformation of three-dimensional chromatin models, and, by this, enhance or suppress gene expression. Through pangenomics datasets of SVs, the fine-tuning of *cis*-elements may bring desirable quantitative traits to breeding programs. Polyploid crops, where multiple copies of a single gene exist, may be of great interest for pangenomics because they modify gene expression through *cis*-elements editing. During a transcriptome time series experiment, Jones et al. [28] investigated spatial-temporal expression of duplicated genes involved in flowering. The *cis*-elements were supposed to create this expression divergence (64% homologous genes of leaf and 74% homologous genes of shoot) and were responsible for the postpolyploidization retentions of selective genes to be expressed or not.

### 4.1. Pangenomics to Identify the Disease Resistance Potential of Crops

In a highly variable crop plant (sunflower), pangenomics was approached to detect genomic variations among 493 different landraces along with wild cultivars [35]. The pangenome analysis exhibited 27% variability among sunflower accessions and out of the 61,205 genes detected in cultivated species, approximately 1.5% of the genes were confirmed for their descendance from the wild germplasm. Of these introgressed genes, the two genes (*SYP132* and GDSL-motif lipase) were found to be related to disease resistance against bacterial and fungal pathogens. Likewise, another study confirmed the role of dispensable genes in plant biotic resistance in rapeseed [36]. The pangenome dataset of 53 rapeseed lines dissected nearly 70% of the variability in dispensable regions between all subjects; however, 50% of these SVs were absent in the cultivated reference ‘*Darmor-bzh*’ [36]. Looking for the resistance genes analogs in different accessions (50 lines) of *Brassica napus,* Dolatabadian et al. [37] identified 753 variable genes out of 1749 total genes related to disease resistance. More specifically, using pangenomics, 368 genes were missing in the datasets generated by mapping to a single reference genome ‘*Darmor-bzh*’. The variable disease resistance genes were rich in SNPs and 106 genes were more specific to blackleg resistance, a highly pathogenic disease of *Brassica* species. The pangenome of *Brachypodium distachyon* generated from 54 accessions revealed the presence of almost a double number of genes compared to previous single genome sequencing [38]. Identification of the disease resistance gene (*Brdisv1Bd1-11011965m*) in the dispensable region was made possible by mapping the lines against the pangenome and the same was missing in the reference genome assembly (Bd21). The ortholog of this resistance gene was found to be up-regulated in wheat-resistant lines against *Puccinia graminis* infections (wheat stem rust) [39].

### 4.2. Pangenomics to Identify the Quantitative Yield Potential of Crops

Food crops with improved yields have remained of great interest to humanity since the beginning of civilization. Staple crops such as wheat and rice have shown great variability among wild and cultivated accessions due to heterozygosity and polyploidy, including paleopolyploidy. A very recent study focusing on wheat improvement [40], developed the wheat graph pangenome to uncover genomic variations (PAV) in different landraces (16 accessions) of bread wheat (*Triticum aestivum* L.). Using the Panache visualization tool, the authors created the first graph pangenome to search SVs for the trait of interest and reported 51,460 missing genes in one cultivar [40]. The graph pangenome of soybean created from 29 representative accessions shortlisted from previous short-read assemblies (2898 accessions) significantly detected SVs among different cultivars [30]. Through GWAS, a 10 kb PAV was detected in the graph pangenome dataset that was related to a hydrophobic protein of the seed cluster in soybean. In another study, the pangenome of pepper (*Capsicum* spp.), constructed from 383 accessions, revealed significant PAV deletions for carotenoid synthesis genes during GWAS [41]. The deletion of a 2.5 kb *Pungent gene 1* fragment found in 50 cultivars was associated with a lower carotenoid content in the pepper fruits. Furthermore, these SVs lead to the formation of yellow or orange fruits in 26 cultivars. The tomato pangenome revealed a lost allele in the flavoring gene of fruit (*TomLoxC*) among cultivated tomatoes that were originally present as SVs in wild tomatoes lying at the promoter of this gene [20]. A rise in the levels of apocarotenoid production in these varieties was accompanied by an inadvertent and unanticipated return of this rare gene in several contemporary elite breeding lines that were first chosen for stress tolerance.

### 4.3. Pangenomics to Develop Climate-Resilient Germplasms

The wild germplasm of many crops has a wide adaptability to variable environments and has shown a relatively higher potential for stress resistance. Domestication with desired traits has narrowed the genomic pools of cultivated crops and made them susceptible to environmental factors. As global climate change threatens agriculture with unpredicted yield losses, it is necessary to restore crop yields to provide food for this ongoing food demand. To explore the genomic diversity of wild progenitors of crop plants, pangenomics offers new insights to capture gene loss and may assist in its recovery, by introgression breeding, or GE, for future crops. For example, stress-responsive genes residing on the dispensable genome have been lost in most crops during domestication, such as soybeans (10.17 to 9.06%) and tomatoes (20.98 to 18.6%) [42]. MAS breeding further narrowed this genomic diversity for cotton and almost every other crop, such as potato, tomato, soybean, wheat, etc. [43]. Breeding bottlenecks involve the selection of germplasms with desired traits while narrowing the genomic diversity at each selection level during the development of modern cultivars [44]. This breeding bottleneck created a limited selection of cultivars for many crops such as maize, cotton, wheat, and barley [45]. However, some crops such as soybean (>45,000 accessions [46]) or potato (>7000 accessions [47]) have huge variability in terms of cultivars diversity. Most of these landraces have been ignored, and only a fraction of this genome diversity is visible in cultivated lines. For example, despite having such variability in soybean germplasms, around 55% of cultivated soybeans in Brazil are comprised of a single cultivar [48] and therefore pose a susceptibility to increased risk of biotic and abiotic stresses. Pangenomics could track this genomic diversity to explore beneficial genes in wild germplasm to be reintroduced into elite cultivars. For example, soybean cultivated species (*Glycine max*) lack resistance genes to sclerotinia stem rot [49], but it was discovered in the wild soybean germplasm. Likewise, the pangenome of rye was analyzed to dissect important genomic information for Triticale improvements [34]. GWAS of the pangenomic datasets (7.86 Gb) of the Chinese rye cultivar (Weining) revealed an expansion of genomic regions that were duplicated for starch biosynthesis, early heading, and gene expression patterns during rye domestication. In cotton, pangenomics datasets built from 1961 accessions revealed a significant set of genes (32,569 and 8851) lost during domestication from wild germplasm [31]. Importantly, most of the lost genes showed PAV landscape in the pangenome assembly and were associated with fiber and yield traits. This PAV of genes may be associated with a loss of germplasm potential to cope with ongoing stresses of climate change and needs to be rectified with pangenomics-assisted breeding.

## 5. Conclusions and Future Prospects

The creation of a graph pangenome is a landmark achievement that has the potential to track the genomic diversity and linkage in cereals with complex and large genomes such as wheat and barley. With extensive effort and capitalization, large pangenomes of cereals are available [50] and could be employed in bioinformatics tools such as Panache [51] for read-mapping and genomics. However, most of the bioinformatics tools available so far have been developed to use the linear format of pangenomes, thus requiring upgradation to input graph pangenomes to capture more information on genomic variations. Further improvements with functional annotations are required to accurately interpret this heritability and genomic variation into some meaningful biological characterization. With the increasing availability of graph pangenomes of different crop species, it becomes possible to compare and track genomic evolutions that could be integrated into breeding programs. The compilation of meta-data from graph pangenomes will enable construction of a genomic map of species-specific traits and could be employed in the synthetic directed evolution of crops. The bridging of pangenomics with pantranscriptomics will be another hallmark with outstanding feasibility to construct species-wide genetic diversity and could revolutionize molecular biology where the expression of specific genes upon diverse biotic and abiotic stresses could be mapped across different germplasms at a time. Recently, some new single cell RNA-Seq technology termed VASA-seq was used to obtain maximum coverage of total RNA in single cell, including non-coding long/short RNAs and non-polyadenylated protein-coding transcripts [52]. In conclusion, graph pangenomes and integrated fast breeding of cereals with higher food value such as enhanced nutritional value and lower mycotoxins accumulation have the potential to cope with the ongoing threats of climate change and food security.

## Figures and Tables

**Table 1:** Trait-associated factors residing on dispensable genomes identified through pangenomics.

Crop	Scientific Name	Pangenome Dataset	Structural Variant	Traits Associated	Number of Accessions	Reference
Rapeseed	*Brassica napus*	1.8 Gb; >150,000 genes	InDels, PAV	Seed weight, flowering, silique length	8	[29]
Soybean	*Glycine max*; *Glycine soja*	57,492 orthologs	PAV	Nutrient uptake	29	[30]
Cotton	*Gossypium hirsutum*; *Gossypium barbadense*	3.3 Gb; >102,000 genes: 2.5 Gb; >80,000 genes	InDels, PAV, SNPs	Disease resistance, fiber quality, stress resistance	1581 for *G. hirsutum*; 226 for *G. barbadense*	[31]
Tomato	*Solanum lycopersicum*	1.1 Gb, 40,369 genes	PAV	Fruit flavor, disease resistance	725	[32]
Maize	*Zea mays*	>103,000 genes	SNPs, PAV, TE, InDels	Flowering; disease resistance	26	[33]
Rye	*Secale cereale*	7.74 Gb; 86,991 genes	TE, Gene duplications	Starch biosynthesis, disease resistance genes	295	[34]
Rice	*Oryza sativa*	1.52 Gb; 51,359 genes	PAV	Grain weight, improved nitrogen uptake	251	[15]
